# Participant and study partner prediction and identification of cognitive impairment in preclinical Alzheimer’s disease: study partner vs. participant accuracy

**DOI:** 10.1186/s13195-019-0539-3

**Published:** 2019-10-18

**Authors:** Mary M. Ryan, Joshua D. Grill, Daniel L. Gillen, for the Alzheimer’s Disease Neuroimaging Initiative

**Affiliations:** 1 Department of Statistics, University of California, Irvine, Irvine, CA 92697 USA; 2 Institute for Memory Impairments and Neurological Disorders, University of California, Irvine, 92697 CA USA; 3 Department of Psychiatry and Human Behavior, University of California, Irvine, Irvine, CA 92697 USA; 4 Department of Neurobiology and Behavior, University of California, Irvine, Irvine, CA 92697 USA; 5 Institute for Clinical and Translational Science, University of California, Irvine, Irvine, CA 92697 USA

**Keywords:** Alzheimer’s disease, Study partner, preclinical, ADNI

## Abstract

**Background:**

Preclinical Alzheimer’s disease (AD) clinical trials require participants to enroll with a study partner, a person who can attend visits and report changes in the participant’s cognitive ability. Whether study partners, compared to participants themselves, provide added information about participant cognition in preclinical AD trials is an open question. We tested the hypothesis that study partners provide meaningful information related to participant cognition cross-sectionally and longitudinally, and assessed whether amyloid status modified observed effects.

**Methods:**

We assessed participant and study partner Everyday Cognition (ECog) scores and participant Alzheimer’s Disease Assessment Scale 13-item cognitive subscale (ADAS13) data from 335 cognitively normal participant-partner dyads in the AD Neuroimaging Initiative. We used random forest and linear mixed effects (LME) models to predict ADAS13 scores as a function of participant and/or study partner ECog scores over time. LME models were adjusted for potential confounding factors, including APOE4 status, amyloid status, baseline age, years of education, and sex. Random forest models were split into the above factors, as well as race/ethnicity and other available neuropsychological battery test scores.

**Results:**

In random forest models predicting ADAS13 12 months from baseline, we observed no difference in the estimated mean variable importance (eMVI) associated with baseline study partner ECog compared to the baseline participant ECog (eMVI = 0.15, 95%CB 0.13, 0.16 for partner; eMVI = 0.15, 95%CB 0.14, 0.16 for participant). In models predicting ADAS13 48 months after baseline, the eMVI associated with baseline study partner ECog was slightly lower than that associated with baseline participant ECog (eMVI = 0.21, 95%CB 0.20, 0.22 for partner; eMVI = 0.24, 95%CB 0.22, 0.25 for participant). In cross-sectional models, study partner eMVI was twice as large as participant eMVI at 12 months (eMVI = 0.20, 95%CB 0.19, 0.21 for partner; eMVI = 0.09, 95%CB 0.09, 0.10 for participant) and three times as large at 48 months (eMVI = 0.38, 95%CB 0.36, 0.39 for partner; eMVI = 0.13, 95%CB 0.12, 0.14 for participant). We did not observe qualitative differences by amyloid status.

**Conclusions:**

While baseline participant reports reasonably predict subsequent cognitive change, informants perform better at cross-sectionally recognizing cognitive status as observation time grows. The study partner requirement may be essential to ensure trial data integrity, especially in longer trials.

## Introduction

Alzheimer’s disease (AD) is a progressive neurodegenerative disease that results in dementia—cognitive and functional impairment that interrupts independence in daily life. US Food and Drug Administration (FDA) approval for new AD treatments historically requires demonstration of both functional and cognitive benefit in AD dementia clinical trials. Demonstration of functional benefit, along with logistical needs related to study compliance and ethical needs related to informed consent, requires that AD dementia trial participants enroll with a study partner [1, 2].

In an effort to intervene earlier in the disease process, before neurodegeneration reaches severe stages, researchers have begun performing clinical trials that enroll patients whose disease does not meet the criteria for dementia. This includes trials of patients with mild cognitive impairment (MCI), a construct defined by objective cognitive impairment without functional loss or impaired activities of daily living. MCI patients with biomarker evidence of AD, such as elevated brain levels of amyloid-β_42_, which can be observed through the use of neuroimaging and cerebrospinal fluid (CSF) protein analysis [3], meet the criteria for *MCI due to AD* or *prodromal AD* [4, 5]. To intervene even earlier in disease, *preclinical AD* trials enroll patients with no cognitive impairment but biomarker evidence of AD [6].

Participants in preclinical and prodromal AD trials are expected to be able to provide informed consent and to comply with study requirements. Yet, the need for informants who report participant cognitive and functional performance in these trials is less understood. Study partners may perform better at predicting future AD dementia than do patients with MCI [7, 8], while other studies show that patients with MCI are fairly accurate at assessing their current cognitive state [9]. Preliminary studies indicate that self-reports from cognitively normal participants may better predict future outcomes than do study partners [10, 11]. Whether these relationships are altered in the presence of AD biomarkers remains an area in need of study.

Initial preclinical AD trials require participants to enroll with study partners [12]. An open question remains, though, whether participants themselves or their partners provide more meaningful information on trial outcomes. In this study, we sought to determine if study partners provide additional information, in relation to preclinical AD study participants, in predicting future cognitive decline or assessing current cognitive performance, and assessed whether amyloid status modified observed effects. We hypothesized that study partners would provide more meaningful information than participants over time, but that this effect would be observed only in participants with elevated brain amyloid.

## Methods

### Data collection

We used data from the AD Neuroimaging Initiative (ADNI). Data used in the preparation of this article were obtained from the ADNI database (adni.loni.usc.edu). The ADNI was launched in 2003 as a public-private partnership, led by principal investigator Michael W. Weiner, MD. The primary goal of ADNI has been to test whether serial magnetic resonance imaging (MRI), positron emission tomography (PET), other biological markers, and clinical and neuropsychological assessment can be combined to measure the progression of MCI and early AD. For up-to-date information, see www.adni-info.org.

Criteria for this study were that participants have a diagnosis of “cognitively normal” at the first visit with an available Everyday Cognition (ECog) score and have at least one observation of amyloid status, as observed via CSF measurement, PET AV45 standard uptake value ratio (SUVR), or PET Pittsburgh compound B (PiB). We used the first visit in which there was an available ECog score as the baseline for this study. After implementing this inclusion/exclusion criteria, there were *N* = 335 viable dyads for the current study: *N* = 227 had data available at a visit 12 months after baseline, *N* = 250 at 24 months, and *N* = 107 at 48 months.

On May 9, 2014, an ADNI2 protocol amendment restricted annual ADNI visits to those participants who progressed to MCI or dementia; cognitively normal participants completed visits every 2 years [13]. The change occurred after all ADNI2 participants were assessed for their 12-month visit, so only month 36 visits were affected. While the protocol change did not impact the entire available data pool, it was significant enough that observed results at 36 months would potentially be biased. Therefore, we excluded any observations collected at 36 months from the current analysis.

### Amyloid positivity

We assigned amyloid status based on CSF amyloid beta (Aβ_42_), AV45 SUVR, or PiB measurements. PiB measurements were converted to the AV45 SUVR scale via the regression equation *y* = 0.15 + 0.67*x* [14]. Those participants with either CSF Aβ_42_ levels below 192 pg/mL or AV45 SUVR (converted or otherwise) above 1.1 were classified as “elevated amyloid”: all others were designated “not elevated amyloid.”

### Alzheimer’s Disease Assessment Scale 13-item cognitive subscale (ADAS13)

We used the Alzheimer Disease Assessment Scale 13-item cognitive subscale (ADAS13) as an objective cognitive performance response variable in this study. Compared to the 11-item version used in dementia trials, the ADAS13 includes a number of cancelation and a delayed free recall task that increases sensitivity in early-stage disease [15, 16]. The ADAS13 has a range of 85 possible points, with higher scores reflecting poorer cognitive performance.

### Everyday Cognition

We used the Everyday Cognition (ECog) scale to examine the subjective cognitive performance in this study. The ECog includes both participant and informant versions. The ECog is a 39-item questionnaire designed to measure functional performance that is linked to specific cognitive abilities [17]. It compares the participant’s current everyday functioning with their perceived functioning (or their study partner’s perception of their functioning) from 10 years prior. Each item covers a task in 1 of 6 cognitively relevant domains (memory, language, visuospatial abilities, planning, organization, and divided attention) and is rated on a 4-point scale, with 1 being “better or no change compared with 10 years earlier” and 4 being “consistently much worse.” Overall ECog scores are calculated by averaging the ratings from the 39 items.

### Statistical methods

Study population demographics were summarized via sample means and standard deviations for continuous variables, and counts and percentages for discrete variables.

We sought to quantify the ability of study participants and partners to cross-sectionally predict cognitive performance and to prospectively predict future changes in cognition, as measured by the ADAS13. We used random forest models to assess the predictive ability of participants and study partners over the course of observation, modeling ADAS13 scores at 12, 24, and 48 months as a function of baseline participant and/or study partner ECog scores [18]. Random forest models represent an ensemble (or average) of Classification and Regression Trees constructed via recursive partitioning where binary separations of the study sample are created by choosing the optimal predictor and cut-point combination to yield the largest discrimination in mean response values between the resulting subpopulations. Random forest models were trained using cross-validation with squared error loss as the prediction penalty, as implemented in the R statistical software language using the GRF package [19, 20]. To account for potential imbalance in the number of repeated measures across subjects, we fit models using a multiple outputation procedure in which a balanced number of observations were sampled from each subject [21]. To maximize efficiency, we took the number of randomly sampled observations per subject to be the minimum number of repeated measures on any one participant in the study sample.

We calculated estimated mean variable importance (eMVI) to assess the relative informative and predictive abilities of participant and study partner ECog scores in the random forest models. Variable importance is measured here as the weighted sum of the frequencies at which a variable is used to split the dataset at various levels—when a variable is used to split the dataset at the top of the decision tree, it is given more weight than when it is used to split the dataset farther down the tree. The larger the variable importance measure, the more important the variable is within the model. To account for stochasticity in the formulation of the random forest models, we created 100 forests by varying the random seed generator in R and calculated the eMVI by taking the sample average of the variable importance measures from each forest. We calculated 95% error bounds by taking the 2.5 and 97.5th percentiles of the simulated variable importance measures.

To provide additional interpretability of the impact of the participant and partner subjective assessment on recognizing participant cognitive decline, we also built linear mixed effects (LME) models on a reduced set of covariates, chosen a priori*.* As with the random forest models, we modeled ADAS13 scores at 12, 24, and 48 months as a function of baseline participant and/or study partner ECog scores. Specifically, we considered models of the form:

$$ \mathrm{ADAS}{13}_{i,j}={\overset{\rightharpoonup }{X}}_{i,0}\overset{\rightharpoonup }{\beta }+{\gamma}_i, $$

where ADAS13_*i*, *j*_ represents the ADAS13 participant score for participant *i* at time point *j*; ${\overset{\rightharpoonup }{X}}_{i,0}$ denotes the vector of baseline covariates for subject *i*, including participant and/or study partner ECog score; $\overset{\rightharpoonup }{\beta }$ represents a vector of fixed effects parameters associated with the baseline covariates; and *γ*_*i*_ denotes a subject-specific random intercept. In addition, all models were adjusted for potential confounding factors, including APOE4 status (carrier vs. non-carrier), amyloid status (elevated vs. not elevated), baseline age, years of education, and sex. Residual diagnostics were conducted to assess the assumption of the exchangeable covariance structure implied by the random intercept model.

To assess the informative ability of participants and study partners at a given time point, we created cross-sectional random forest and LME models using ECog scores at 12, 24, and 48 months to predict ADAS13 scores at the same time points. We used a similar approach as above, except baseline ECog scores were replaced with concurrent scores for the visit at which the response was obtained.

## Results

Table 1 describes the demographics of the participants included in this analysis, stratified by amyloid status. Compared to the not elevated group, the elevated amyloid group included more female participants (77% vs. 47%) and more participants with at least one ε4 allele of the APOE gene (47% vs. 23%). Both groups had similar mean ages, years of education, and participant and study partner baseline ECog scores, though the elevated amyloid group had higher baseline ADAS13 scores (9.63 vs. 8.65). In both the elevated and the not elevated amyloid groups, participants scored their cognitive function on the ECog worse than did their study partners at baseline. Demographic data for ADNI study partners were not available.

**Table 1:** Characteristics of participants and study partners analyzed

	Amyloid beta elevated (*n* = 100)	Amyloid beta not elevated (*n* = 235)	Total (*n* = 335)
Sex			
Male	33 (33%)	124 (52.77%)	157 (46.87%)
Female	77 (77%)	111 (47.23%)	178 (53.13%)
Race/ethnicity			
Caucasian	89 (89%)	212 (90.21%)	301 (89.85%)
African-American	7 (7%)	15 (6.38%)	22 (6.57%)
Asian	2 (2%)	4 (1.7%)	6 (1.79%)
Age	74.34 (5.87)	72.8 (5.77)	73.26 (5.83)
Years of education	15.93 (2.56)	16.72 (2.55)	16.49 (2.58)
APOE4 alleles			
0	53 (53%)	182 (77.45%)	235 (70.15%)
1	43 (43%)	49 (20.85%)	92 (27.46%)
2	4 (4%)	4 (1.70%)	8 (2.39%)
MMSE	29.04 (1.08)	29.08 (1.28)	29.07 (1.22)
ADAS13	9.63 (4.43)	8.65 (4.42)	8.94 (4.44)
ECog			
Participant	1.42 (0.32)	1.38 (0.33)	1.39 (0.33)
Study partner	1.21 (0.29)	1.20 (0.30)	1.20 (0.29)

**Fig. 1:**
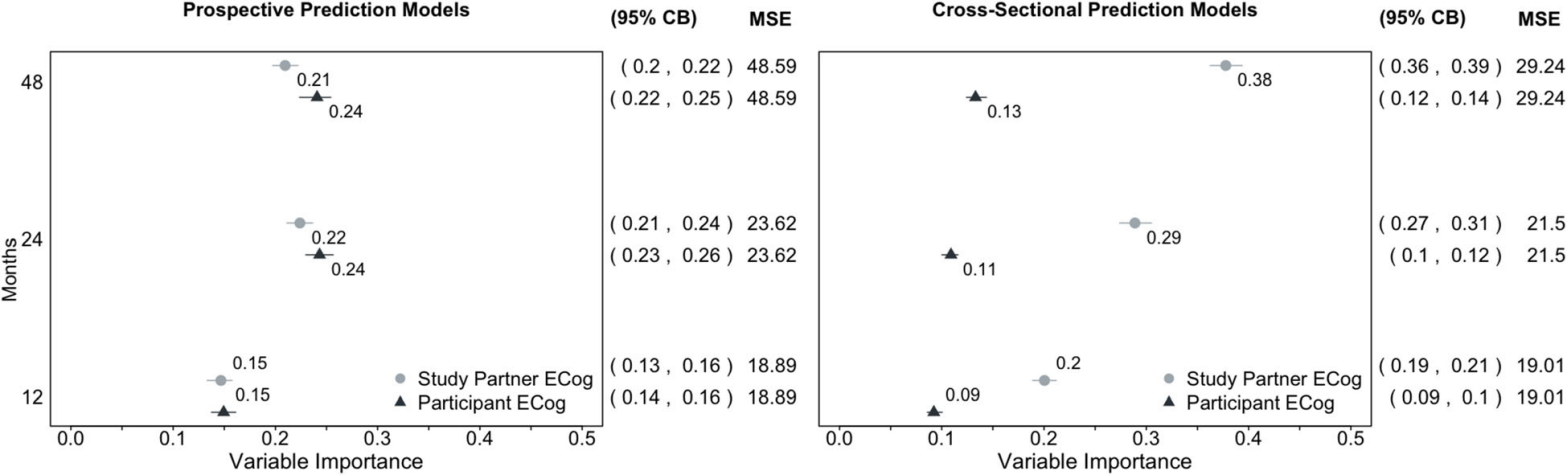
Variable importance of baseline (**a**) and 12-, 24-, and 48-month (**b**) participant and study partner ECog scores predicting 12-, 24-, and 48-month ADAS13 scores in the additive model using the same covariates as in the LME models

In random forest models predicting ADAS13 12 months from baseline, the eMVI associated with baseline study partner ECog was not different from that associated with baseline participant ECog (eMVI = 0.15, 95%CB 0.13, 0.16 for partner; eMVI = 0.15, 95%CB 0.14, 0.16 for participant; Fig. 1a). Similarly, when predicting ADAS13 further from baseline to 48 months, the eMVI associated with baseline study partner ECog was slightly lower than that associated with baseline participant ECog (eMVI = 0.21, 95%CB 0.20, 0.22 for partner; eMVI = 0.24, 95%CB 0.22, 0.25 for participant; Fig. 1a). These results were mirrored in the longitudinal LME models predicting the same time points (see Tables 2 and 3).

**Table 2:** LME coefficients for longitudinal and cross-sectional models at 12 months from baseline

	Longitudinal model		Cross-sectional model	
	Coefficient estimate	95% confidence interval	Coefficient estimate	95% confidence interval
Participant ECog	0.16	(− 0.50, 0.79)	0.06	(− 0.44, 0.56)
Study partner ECog	0.72	(0.07, 1.36)	0.67	(0.11, 1.23)
Time	− 1.22	(− 184.30, 181.86)	− 14.02	(− 200.75, 172.71)
Squared time	3.17	(− 89.14, 95.48)	9.72	(− 84.41, 103.86)
Age	0.18	(0.08, 0.27)	0.17	(0.07, 0.27)
Years of education	− 0.27	(− 0.49, − 0.06)	− 0.27	(− 0.50, − 0.05)
Male	1.73	(0.60, 2.87)	1.78	(0.63, 2.94)
> 0 APOE4	0.61	(− 0.62, 1.85)	0.56	(− 0.71, 1.83)
Amyloid status	− 0.10	(− 1.41, 1.20)	− 0.04	(− 1.37, 1.30)

**Table 3:** LME coefficients for longitudinal and cross-sectional models at 48 months from baseline

	Longitudinal model		Cross-sectional model	
	Coefficient estimate	95% confidence interval	Coefficient estimate	95% confidence interval
Participant ECog	0.24	(− 1.06, 1.54)	0.66	(− 0.17, 1.48)
Study partner ECog	1.18	(− 0.39, 2.75)	1.37	(0.78, 1.96)
Time	− 546.09	(− 1469.04, 376.87)	− 526.34	(− 1397.70, 345.01)
Squared time	70.25	(− 45.59, 186.1)	68.18	(− 41.18, 177.53)
Age	0.25	(0.05, 0.45)	0.21	(0.02, 0.40)
Years of education	− 0.36	(− 0.80, 0.08)	− 0.38	(− 0.80, 0.03)
Male	1.10	(− 1.12, 3.31)	1.01	(− 1.10, 3.11)
> 0 APOE4	1.65	(− 0.63, 3.94)	1.29	(− 0.86, 3.43)
Amyloid status	1.58	(− 0.76, 3.91)	0.83	(− 1.43, 3.08)

In cross-sectional random forest models assessing 12-month ADAS13, the eMVI associated with study partner ECog at 12 months was twice as large as the eMVI associated with participant ECog at 12 months (eMVI = 0.20, 95%CB 0.19, 0.21 for partner; eMVI = 0.09 95%CB 0.09, 0.10 for participant; Fig. 1b). By 48 months, the eMVI associated with study partner ECog for assessing ADAS13 was three times as large as that associated with participant ECog—a statistically significant difference (eMVI = 0.38, 95%CB 0.36, 0.39 for partner; eMVI = 0.13, 95%CB 0.12, 0.14 for participant; Fig. 1b).

A similar gap was observed at 48 months in the cross-sectional LME model: a 1 standard deviation increase in study partner ECog was associated with a 1.37-point increase in ADAS13 score (95% CI 0.78, 1.96; Table 3) while a 1 standard deviation increase in participant ECog was associated with a 0.66-point increase (95% CI − 0.17, 1.48; Table 3). Removing cases of conversion to MCI from the analyses had minimal impact at 12 and 24 months but essentially removed the observed difference in eMVI between partners and participants at 48 months (data not shown).

Across all LME models—including those predicting future ADAS13 scores from baseline ECog scores and cross-sectional models—the effect of amyloid status was not statistically significant. Likewise, there were there no significant interacting effects between amyloid status and participant or study partner ECog scores in any LME model.

## Discussion

We investigated the extent to which study partners provide relevant additional information, in relation to study participants, in a sample that parallels preclinical AD trials. While eMVI values cannot be compared across models, because the outcomes measured in each model are different and hence yield inherently different variation, we can compare the relative performance of participants and study partners within each setting. When we assessed the participants’ and partners’ abilities at baseline to predict future cognitive performance through random forest models, we found no difference between the groups. When examining the relationships between participant and partner reporting cross-sectionally, however, we found that the study partners provide increasingly meaningful information over time and that this information eventually (by 48 months) becomes more valuable than the participant’s self-report. Similar results were seen in the associative LME models: higher study partner ECog scores were associated with higher ADAS13 scores, compared to ECog scores provided by participants, in both longitudinal and cross-sectional models. In the prospective prediction case, the width of this gap stayed approximately static over time, whereas in cross-sectional models, this gap widened over time.

These results are similar to the previous observations and indicate that the information provided by the study partners becomes increasingly important over the course of a preclinical AD trial. For example, Amariglio and colleagues observed higher correlations between participant Cognitive Function Instrument (CFI) scores and a composite cognitive outcome at baseline, compared to study partner CFI scores. By 48 months, however, correlations were higher for partners compared to participants, though summing CFI for the dyad demonstrated the highest correlations [11]. Two, not mutually exclusive, potential explanations exist for why study partners may increasingly outperform participants in recognizing cognitive impairment in preclinical AD trials. First, the quality of the information provided by the study partner may increase with time, as they become more comfortable with the instrument, study procedures, and the need to observe participant behaviors between visits. Second, some participants may experience cognitive decline over the course of the study, rendering them less able to reliably report their own cognitive and functional performance. For example, a portion of MCI participants may demonstrate anosognosia, and these individuals may be at the highest risk for AD and progression to dementia [22, 23].

Notably, a minority (*N* = 47) of the participants in this study progressed to a clinical diagnosis of MCI. When removing those who converted to MCI at the 48-month time point, the observed cross-sectional effect disappeared. Partners’ eMVI were no different from participants’. Thus, conversion to MCI is largely driving the observed overall effects at 48 months. Though this is important, it does not change the overall interpretation of our results. Indeed, preclinical AD trials aim to enrich for participants most likely to demonstrate cognitive decline during the course of study, ultimately including the onset of MCI or dementia in some participants. This is very much in line with the primary aim of these studies: to enable the efficient assessment of whether an intervention can delay or prevent the onset of cognitive impairment.

In contrast to our hypothesis, we did not observe differences in the associations between subjective reports and objective performance based on amyloid status. The length of the asymptomatic period of AD has been estimated to be as long as 15 years [24]. Recent demonstrations of the differences in cognitive performance between those with elevated and not elevated brain amyloid were most striking more than 48 months after baseline, beyond the length of longitudinal data used in the current study [25]. We chose this length of longitudinal assessment to recapitulate the lengths of preclinical AD trials, and also because of the limited data available based on our study design (i.e., the use of the ECog as a subjective assessment of participant cognitive performance). The risk for cognitive decline may also be highest in individuals who not only demonstrate elevated amyloid, but who are also carriers for the ε4 allele of the APOE gene, and we lacked sufficient sample size to permit such further stratification in our analyses [26].

Amyloid status was positively associated with ADAS13 48 months from baseline but was not statistically significant due to high variation in the observed estimate. Predictors of ADAS13 scores (cross-sectionally and longitudinally) included age (positive association), education (negative association), and male sex (positive association). The effects of age and education are not surprising. The effect of sex, however, was unexpected, given that women are at increased risk for AD [27]. Men are, however, at increased risk for MCI and sex effects may yield differences not only in temporal progression of disease, but also specific cognitive domains that are affected early vs. late in disease [28, 29].

### Limitations

As is the case with any observational study, there is a possibility that the relative informative ability of the study partners is affected by factors that were not observed. We attempted to mitigate this to the best of our ability by controlling for known confounding factors but were limited to the data related to the participants themselves. No data were available for the study partners. Characteristics such as whether the study partner lives with the participant or the number of hours spent per week in contact with the participant may affect the quality of data they provide [30], and an accompanying paper by our group examines this concept in preclinical AD trials [31]. In addition, due to protocol amendments in ADNI2, reliable data were not available at 36 months. We have no reason to suspect, however, that estimates would stray from the observed trajectories created by the 12-, 24-, and 48-month models. Moreover, there were few viable dyads remaining for 48-month analyses, limiting the estimate precision at this time point. More information at these time points may be necessary to credibly confirm the inferences made in this study.

## Conclusions

While cognitively normal participants may be capable of providing consent and accurately informing on their own cognitive abilities at study start, study partner information is likely to become increasingly important over the course of a preclinical AD trial. Given that clinical trials tend to focus on current participant cognitive status measured prospectively in time, the finding that study partners provide increasingly more predictive data for assessing participant cognition as time moves on has potentially far-reaching implications in the setting of controlled intervention trials. Specifically, this finding suggests that the study partner role may be essential to minimizing bias, increasing precision in endpoint assessment, and ultimately ensuring trial data integrity. Thus, these results endorse the continued requirement of study partners in preclinical AD trials.

## Data Availability

Not applicable.

## Abbreviations

Aβ_42_: amyloid beta
AD: Alzheimer’s disease
ADAS13: Alzheimer Disease Assessment Scale 13-item cognitive subscale
ADNI: Alzheimer’s Disease Neuroimaging Initiative
ADNI2: Second phase of ADNI
CB: Confidence bound
CI: Confidence interval
CSF: Cerebrospinal fluid
ECog: Everyday Cognition
eMVI: Estimated mean variable importance
FDA: Federal Drug Administration
LME: Linear mixed effects
MCI: Mild cognitive impairment
MRI: Magnetic resonance imaging
PET: Positron emission tomography
PiB: Pittsburgh compound B
SUVR: Standard uptake value ratio

## References

[1] U.S. Department of Health and Human Services, Food and Drug Administration, Center for Drug Evaluation and Research (CDER), Center for Biologics Evaluation and Research (CBER) (2018). Early Alzheimer’s disease: developing drugs for treatment guidance for industry - draft guidance.

[2] Largent EA, Karlawish J, Grill JD (2018). Study partners: essential collaborators in discovering treatments for Alzheimer’s disease. Alzheimers Res Ther.

[3] Hardy JA, Higgins GA (1992). Alzheimer’s disease: the amyloid cascade hypothesis. Science.

[4] Albert MS, DeKosky ST, Dickson D, Dubois B, Feldman HH, Fox NC (2011). The diagnosis of mild cognitive impairment due to Alzheimer’s disease: recommendations from the National Institute on Aging-Alzheimer’s Association workgroups on diagnostic guidelines for Alzheimer’s disease. Alzheimers Dement.

[5] Dubois B, Feldman HH, Jacova C, Cummings JL, DeKosky ST, Barberger-Gateau P (2010). Revising the definition of Alzheimer’s disease: a new lexicon. Lancet Neurol.

[6] Sperling RA, Aisen PS, Beckett LA, Bennett DA, Craft S, Fagan AM (2011). Toward defining the preclinical stages of Alzheimer’s disease: recommendations from the National Institute on Aging-Alzheimer’s Association workgroups on diagnostic guidelines for Alzheimer’s disease. Alzheimers Dement.

[7] Tierney MC, Szalai JP, Snow WG, Fisher RH (1996). The prediction of Alzheimer disease: the role of patient and informant perceptions of cognitive deficits. Arch Neurol.

[8] Tierney MC, Herrmann N, Geslani DM, Szalai JP (2003). Contribution of informant and patient ratings to the accuracy of the Mini-Mental State Examination in predicting probable Alzheimer’s disease. J Am Geriatr Soc.

[9] Piras F, Piras F, Orfei MD, Caltagirone C, Spalletta G (2016). Self-awareness in mild cognitive impairment: quantitative evidence from systematic review and meta-analysis. Neurosci Biobehav Rev.

[10] Farias ST, Lau K, Harvey D, Denny KG, Barba C, Mefford AN (2017). Early functional limitations in cognitively normal older adults predict diagnostic conversion to mild cognitive impairment. J Am Geriatr Soc.

[11] Amariglio RE, Donohue MC, Marshall GA, Rentz DM, Salmon DP, Ferris SH (2015). Tracking early decline in cognitive function in older individuals at risk for Alzheimer disease dementia: the Alzheimer’s Disease Cooperative Study Cognitive Function Instrument. JAMA Neurol.

[12] Grill JD, Karlawish J (2017). Study partners should be required in preclinical Alzheimer’s disease trials. Alzheimers Res Ther.

[13] Albert M, DeKosky S, Salmon D, Morris J, Cairns N. Alzheimer’s disease neuroimaging initiative 2 (adni2) protocol (ADC-039). 2015;59.

[14] Landau SM, Breault C, Joshi AD, Pontecorvo M, Mathis CA, Jagust WJ (2013). Amyloid-β imaging with Pittsburgh compound B and florbetapir: comparing radiotracers and quantification methods. J Nucl Med.

[15] Mohs RC, Knopman D, Petersen RC, Ferris SH, Ernesto C, Grundman M (1997). Development of cognitive instruments for use in clinical trials of antidementia drugs: additions to the Alzheimer’s Disease Assessment Scale that broaden its scope. The Alzheimer’s Disease Cooperative Study. Alzheimer Dis Assoc Disord.

[16] Sano M, Raman R, Emond J, Thomas RG, Petersen R, Schneider LS (2011). Adding delayed recall to the Alzheimer Disease Assessment Scale is useful in studies of mild cognitive impairment but not Alzheimer disease. Alzheimer Dis Assoc Disord.

[17] Farias ST, Mungas D, Reed BR, Cahn-Weiner D, Jagust W, Baynes K (2008). The measurement of Everyday Cognition (ECog): scale development and psychometric properties. Neuropsychology.

[18] Breiman L (2001). Random forests. Mach Learn.

[19] R Core Team (2017). R: a language and environment for statistical computing.

[20] Tibshirani J, Athey S, Wager S, Friedberg R, Miner L, Wright M (2018). grf: generalized random forests (beta).

[21] Follmann D, Proschan M, Leifer E (2003). Multiple outputation: inference for complex clustered data by averaging analyses from independent data. Biometrics.

[22] Munro CE, Donovan NJ, Amariglio RE, Papp KV, Marshall GA, Rentz DM (2018). The impact of awareness of and concern about memory performance on the prediction of progression from mild cognitive impairment to Alzheimer disease dementia. Am J Geriatr Psychiatry.

[23] Therriault J, Ng KP, Pascoal TA, Mathotaarachchi S, Kang MS, Struyfs H (2018). Anosognosia predicts default mode network hypometabolism and clinical progression to dementia. Neurology.

[24] Sperling R, Mormino E, Johnson K (2014). The evolution of preclinical Alzheimer’s disease: implications for prevention trials. Neuron.

[25] Donohue MC, Sperling RA, Petersen R, Sun C-K, Weiner MW, Aisen PS (2017). Association between elevated brain amyloid and subsequent cognitive decline among cognitively normal persons. JAMA.

[26] Lim YY, Kalinowski P, Pietrzak RH, Laws SM, Burnham SC, Ames D (2018). Association of β-amyloid and apolipoprotein E ε4 with memory decline in preclinical Alzheimer disease. JAMA Neurol.

[27] Mielke MM, Vemuri P, Rocca WA (2014). Clinical epidemiology of Alzheimer’s disease: assessing sex and gender differences. Clin Epidemiol.

[28] Petersen RC, Roberts RO, Knopman DS, Geda YE, Cha RH, Pankratz VS (2010). Prevalence of mild cognitive impairment is higher in men: the Mayo Clinic Study of Aging. Neurology.

[29] Sundermann EE, Biegon A, Rubin LH, Lipton RB, Mowrey W, Landau S (2016). Better verbal memory in women than men in MCI despite similar levels of hippocampal atrophy. Neurology.

[30] Ready R, Ott B, Grace J (2004). Validity of informant reports about AD and MCI patients’ memory. Alzheimer Dis Assoc Disord.

[31] Nuño MM, Gillen DL, Grill JD. Study Partner Types and Prediction of Cognitive Performance: Implications to Pre-Clinical Alzheimer’s Trials. Alzheimers Res Ther. In press 10.1186/s13195-019-0544-6 PMC6881999 31775871

